# Assessing the Content and Effect of Web-Based Decision Aids for Postmastectomy Breast Reconstruction: Systematic Review and Meta-Analysis of Randomized Controlled Trials

**DOI:** 10.2196/53872

**Published:** 2024-05-27

**Authors:** Lin Yu, Jianmei Gong, Xiaoting Sun, Min Zang, Lei Liu, Shengmiao Yu

**Affiliations:** 1 School of Nursing, Liaoning University of Chinese Traditional Medicine Shenyang China; 2 Outpatient Department, The Fourth Affiliated Hospital of China Medical University Shenyang China

**Keywords:** decision aids, internet, postmastectomy breast reconstruction, decision conflicts, mobile phone

## Abstract

**Background:**

Web-based decision aids have been shown to have a positive effect when used to improve the quality of decision-making for women facing postmastectomy breast reconstruction (PMBR). However, the existing findings regarding these interventions are still incongruent, and the overall effect is unclear.

**Objective:**

We aimed to assess the content of web-based decision aids and its impact on decision-related outcomes (ie, decision conflict, decision regret, informed choice, and knowledge), psychological-related outcomes (ie, satisfaction and anxiety), and surgical decision-making in women facing PMBR.

**Methods:**

This systematic review and meta-analysis followed the PRISMA (Preferred Reporting Items for Systematic Reviews and Meta-Analyses) guidelines. A total of 6 databases, PubMed, Embase, Cochrane Library, CINAHL, PsycINFO, and Web of Science Core Collection, were searched starting at the time of establishment of the databases to May 2023, and an updated search was conducted on April 1, 2024. MeSH (Medical Subject Headings) terms and text words were used. The Cochrane Risk of Bias Tool for randomized controlled trials was used to assess the risk of bias. The certainty of evidence was assessed using the Grading of Recommendations, Assessment, Development, and Evaluation approach.

**Results:**

In total, 7 studies included 579 women and were published between 2008 and 2023, and the sample size in each study ranged from 26 to 222. The results showed that web-based decision aids used audio and video to present the pros and cons of PMBR versus no PMBR, implants versus flaps, and immediate versus delayed PMBR and the appearance and feel of the PMBR results and the expected recovery time with photographs of actual patients. Web-based decision aids help improve PMBR knowledge, decisional conflict (mean difference [MD]=–5.43, 95% CI –8.87 to –1.99; *P*=.002), and satisfaction (standardized MD=0.48, 95% CI 0.00 to 0.95; *P*=.05) but have no effect on informed choice (MD=–2.80, 95% CI –8.54 to 2.94; *P*=.34), decision regret (MD=–1.55, 95% CI –6.00 to 2.90 *P*=.49), or anxiety (standardized MD=0.04, 95% CI –0.50 to 0.58; *P*=.88). The overall Grading of Recommendations, Assessment, Development, and Evaluation quality of the evidence was low.

**Conclusions:**

The findings suggest that the web-based decision aids provide a modern, low-cost, and high dissemination rate effective method to promote the improved quality of decision-making in women undergoing PMBR.

**Trial Registration:**

PROSPERO CRD42023450496; https://www.crd.york.ac.uk/prospero/display_record.php?RecordID=450496

## Introduction

### Background

Breast cancer (BC) is a major global health problem. In 2020, more than 2.3 million newly diagnosed cases and 685,000 deaths were associated with BC [1]. There has been a gradual increase in the incidence of BC globally over the past few decades, which has been attributed to lifestyle changes (eg, increase in BMI and decrease in birth rate), as well as an increase in screening detection as BC becomes more recognized [2-4]. Although BC has the highest incidence rate among all types of cancer, its mortality rate declined by 43% between 1989 and 2020, and it is concentrated in larger areas [2,5]. Advances in the early detection and treatment of BC have improved patient survival rates, which, in turn, have led to an increased focus on improving the quality of life of the survivors of BC. The surgical approach to BC is complex and usually involves the decision to undergo breast-conserving surgery and mastectomy. For women undergoing mastectomy, the change in appearance due to the missing breast can lead to various types of psychological problems including physical imagery discomfort, psychological distress, anxiety, and depression [6]. Postmastectomy breast reconstruction (PMBR) is now an option for women to restore their appearance [7].

However, when women face a PMBR decision, they must decide whether to use PMBR, and if they choose to do so, they should further decide on the timing and type of PMBR (ie, implant, autologous tissue, or combination) [8,9]. Delayed autologous PMBR results in a localized or regional recurrence rate similar to immediate PMBR [10]. A BC diagnosis can leave patients feeling anxious and uncertain, which is often exacerbated by presenting multiple, complex treatment options for women to choose from in a short period [11]. Most patients with BC who are considering PMBR immediately have clinically substantially decision conflict [12,13]. Patients experience postoperative complications leading to decision regret [14]. These issues can lead to poorer health outcomes, negative perceptions of the health care system, and lower quality of life [14,15]. Therefore, more preoperative patient education about possible complications includes the patient’s anatomy, which PMBR to choose, the associated pros and cons, and previous surgical and medication history. Women should be fully informed of their options and given the tools to weigh the pros and cons of each option, which may reduce the incidence of these adverse effects [16]. At the same time, personalized medicine is increasingly becoming the standard of care for patients with BC [17], and based on the current evidence, patients should have equal access to all eligible PMBR options [10]. In a sample of 126 patients who underwent mastectomy, a minority of patients made high-quality decisions about PMBR. Specifically, 43.3% of patients were adequately informed and accepted treatment decisions that were consistent with their preferences [11]. Therefore, patients and providers must work together through dialogue to optimize treatment options and engage in shared decision-making. However, it is not easy for inexperienced physicians to perform shared decision-making in an orderly and correct manner in a limited amount of time [18]. Decision aids may be helpful before a patient decides to undergo PMBR. Some studies [19] also suggest that decision aids may be helpful for some women even after undergoing a PMBR, as some women exhibit decision conflicts after the consultation. Decision aids are powerful tools to support patients in making informed choices based on their own values and are available via the internet, DVDs, and printed materials [20]. With the increasing popularity of the internet worldwide, web-based dissemination of information has been recognized as one of the most promising of all available formats (eg, leaflets, brochures, audio, and video) for delivering decision aids to patients. Web-based decision aids are characterized as being interactive, dynamic, and customizable [21]. On the one hand, web-based decision aids have a greater advantage in facilitating patient access than face-to-face interaction with physicians. On the other hand, decision aids on the internet can store and disseminate information over a longer period than traditional, static decision aids and can personalize the visit according to the patient’s values and preferences [21-23].

### Prior Research

Paraskeva et al [24] conducted a systematic review exploring the effectiveness of interventions to assist women in making decisions about PMBR, which consisted of 6 studies with mixed results in terms of knowledge, decision-making, overall satisfaction, and quality of life. Berlin et al [25] assessed PMBR decision aids in a systematic review and meta-analysis, concluding that PMBR reduces decision conflict, improves information satisfaction, promotes participation in the decision-making process, and enhances the awareness of participation in the decision-making process. However, the authors included all types of trials (ie, quantitative and qualitative) and only meta-analyzed decision conflict. This review also did not include the effects of decision aids on outcome indicators such as psychologically relevant outcomes. Yang et al [26] conducted a meta-analysis exploring the effects of decision aids on decision-making in PMBR; however, the authors did not compare whether different forms of decision aids would have different effects. Zhao et al [27] conducted a scoping review with the aim of reviewing, comparing, and discussing the current incorporation of the adverse effects of BC treatments into decision aids and examined how web-based decision aids personalized BC treatment decision-making tools in patient–health care provider communication, clinician decision-making processes, and shared decision-making, as yet unassessed patient outcomes (eg, knowledge and anxiety). In summary, there is a lack of descriptions of the impact of web-based decision aids on the decision-making of women facing PMBR. Overall, existing systematic evaluations on related topics have produced mixed results, and more importantly, many primary trials [28-31], following these reviews, have produced conflicting results, which may provide new evidence. Therefore, there is a need for a new systematic evaluation to provide a comprehensive overview of the effectiveness of web-based decision aids on the quality of decision-making for women faced with PMBR, drawn from all available evidence from randomized controlled trials (RCTs) that meet high standards for evidence-based research.

### Objectives

The aim of this systematic review and meta-analysis was to assess the content of the web-based decision aids and evaluate their effectiveness on decision-related outcomes (ie, decision conflict, decision regret, informed choice, and knowledge), psychological-related outcomes (ie, satisfaction and anxiety), and surgical decision-making in women facing PMBR.

## Methods

This is a systematic review and meta-analysis reported in accordance with PRISMA (Preferred Reporting Items for Systematic Reviews and Meta-Analyses; Multimedia Appendix 1) guidelines [32]. The protocol was registered in PROSPERO (CRD42023450496).

### Eligibility and Exclusion Criteria

An overview of the inclusion and exclusion criteria can be found in Textbox 1.

**Eligibility and exclusion criteria.**

**Population**

- The population included in the study was aged ≥18 years and women who had been diagnosed with breast cancer (BC) and were considering postmastectomy breast reconstruction (PMBR) but had not yet had the surgery and had internet access. If the patient, at the time of enrollment, had attempted PMBR; did not have BC (ie, were considering prophylactic mastectomy); and had an active psychiatric, cognitive, or visual impairment, they were not eligible.

**Intervention**

- Studies focusing on web-based decision aids (including websites and apps)

**Comparison**

- Controls for usual care, counseling, health education pamphlets, and non–web-based decision aids

**Outcome**

- The primary outcomes were decision-related outcomes (ie, informed choice, knowledge, decision conflict, and decision regret); psychological outcomes (ie, satisfaction and anxiety); and PMBR options and tool usability (ie, women’s feedback on use)

**Study**

- Randomized controlled trials

### Search Strategy

A systematic search of studies was carried out using English databases such as PubMed, Embase, Cochrane Library, CINAHL, PsycINFO, and Web of Science Core Collection from the date of inception of each database to May 2023, and an updated search was conducted on April 1, 2024, to cover new research. Medical Subject Headings terms and text words were used. The keywords used included “Mastectomy,” “mammaplasty,” “mastectomy,” “informed choice*,” “shared decision making,” “computer,” “web based,” and “Internet,” which are English search terms. These index terms and keywords were explored and modified according to the different grammatical rules of the database. Specific details of the search algorithm are available in Multimedia Appendix 2. The reference lists of the included studies and relevant articles were hand-searched to identify other potentially eligible articles. The search was limited to articles in English and had no limitations with regard to publication year.

### Screening

The results were input into EndNote X9, and duplicates were removed automatically. After removing duplicates, 2 reviewers independently screened the titles and abstracts of identified articles and removed irrelevant citations in accordance with the selection criteria. After the removal of irrelevant studies, the full texts of potentially relevant studies were retrieved. Next, both reviewers independently assessed the full texts. Any disagreements were settled by discussion with a third reviewer.

### Data Extraction

Characteristics of the included RCTs (eg, author, year of publication, country, sample size, subject characteristics, form, content, development method and team, theoretical basis, duration of use, reading level, a brief description of the intervention in the control group, outcome measurements, follow-up, and results) were extracted into tables. We wrote to the authors to obtain more information about the results. Two reviewers compared the findings independently.

### Risk of Bias Assessment

The quality of RCTs was evaluated using the Cochrane Handbook for RCTs [33]. The tool consists of 7 items: randomized sequence generation, allocation concealment, participant and personnel blinding, blinding for outcome assessment, incomplete outcome, data selective reporting, and other bias. The risk of bias for each domain was judged as low risk of bias, high risk of bias, or unclear risk of bias. The evaluation of study quality was performed independently by 2 reviewers, and a third reviewer was consulted if necessary.

### Statistical Analysis

Statistical analysis was performed using Review Manager (version 5.3; Cochrane), illustrated using a forest plot when at least 2 studies were measured for the same outcomes for a PMBR decision at the longest follow-up time point [34,35]. We used mean differences (MDs) for continuous variables that were measured with the same instrument, standardized MDs (SMDs) when a similar outcome was assessed with different instruments, and relative risks for dichotomous variables. We calculated possible missing values such as SD and 95% CI [33]. In the study, heterogeneity was assessed via the Higgins *I*^2^ statistic with *I*^2^ values of ≤25%, 50%, and ≥75% deemed to represent low, medium, and high heterogeneity, respectively [33]. When there was no significant heterogeneity, the fixed effects model (*I*^2^≤50%) was used; otherwise, the random effects model was used, resulting in a more conservative summary effect estimate [33]. To identify potential sources of clinical heterogeneity, we also conducted a post hoc sensitivity analysis to determine the stability of the results by omitting each test [36].

## Results

### Study Selection

**Figure 1:**
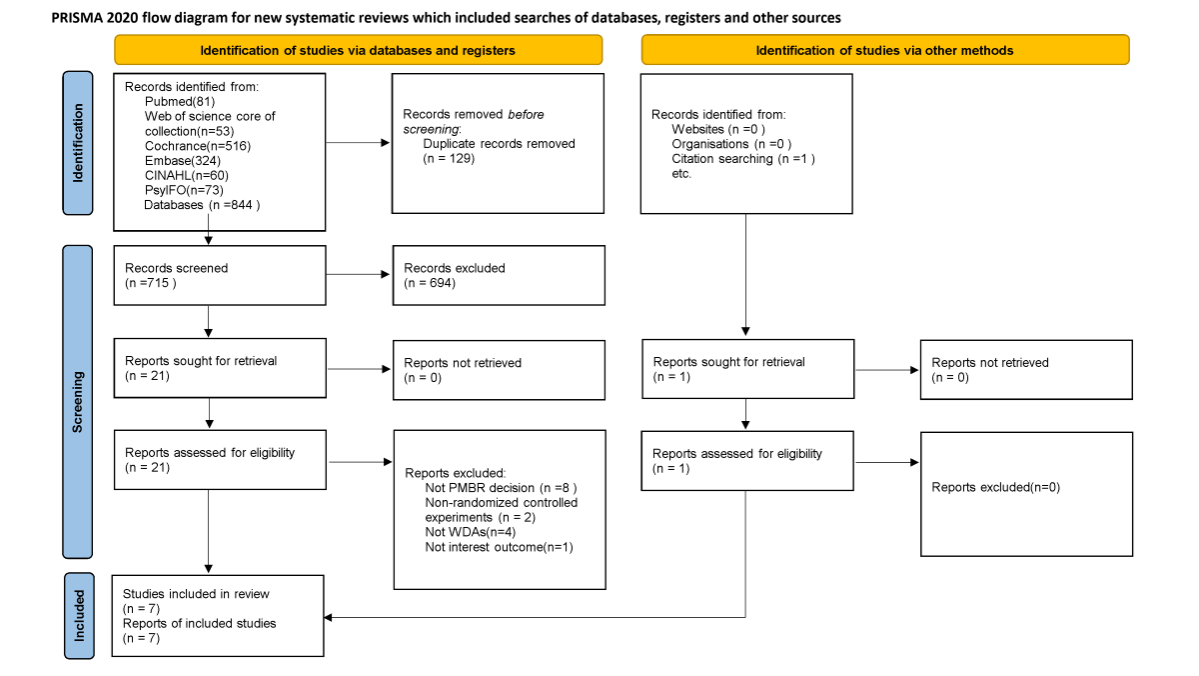
PRISMA (Preferred Reporting Items for Systematic Reviews and Meta-Analyses) flow diagram.

Figure 1 shows the research selection process and results based on the PRISMA 2020 guidelines. A total of 844 studies were identified. A total of 129 of these studies were excluded because they were repetitive. After selecting titles and abstracts, 21 studies were included for the next stage. Consequently, 7 studies met the inclusion criteria.

### Study Characteristics

The 7 studies included 579 women and were published between 2008 and 2023, and the sample size in each study ranged from 26 to 222. The average age of the women was approximately 50 years; they were in the early stages of BC and facing the PMBR decision. The studies were conducted in 3 countries; 6 studies were conducted in high-income countries—4 in the United States [30,31,37,38] and 2 in Australia [29,39]—and 1 in an upper–middle-income country, China [28]. Detailed characteristics of the included studies are shown in Table 1.

**Table 1:** Basic characteristics of the included studies.

Study and country	Population	Sample size, n			Outcome measures		Timing		Outcome
		Experimental	Control						
Fang et al [28], China	Aged ≥20 years, recently diagnosed with BC^a^, candidates for mastectomy, and able to read and speak Taiwanese or Mandarin	48	48	DCS^b^, BR-DMPS^c^, DRS^d^, BIS^e^, and HADS^f^		1 week, 1.8 months, and 12 months		Understanding of medical information, DC^g^, anxiety, depression, DR^h^, and body image distress	
Heller et al [37], United States	Unable to read, write, and speak English, had previously viewed the interactive digital education aid, and the initial consultation was for the purpose of obtaining a second opinion	66	67	Knowledge and satisfaction (self-administered scales) and STAI^i^		1 month after surgery		Knowledge, satisfaction, acquiring information about BR^j^, and anxiety	
Manne et al [38], United States	Women, aged >18 years, English speaking, diagnosed with ductal carcinoma in situ or stage 1, 2, or 3a BC, considering mastectomy but had not yet had the surgery and had home internet access or willingness to use the patient education center computer to access the web-based decision aids, if assigned to this condition	31	24	Knowledge and satisfaction (self-administered scales), DCS, and STAI		2 weeks		BR knowledge, preparation to make a decision, DC, anxiety, and BR intentions	
Mardinger et al [29], Australia	Planning to undergo or having already undergone a therapeutic or prophylactic mastectomy, considering BR, aged >18 years, a proficient in English, and with internet access	30	30	DCS, DRS, and the Satisfaction with Decision Scale		6 weeks and 6 months		Satisfaction with decision, DC, and DR	
Politi et al [30], United States	Adult (≥18 years) English-speaking women with stages 0-III BC, who were considering a referral or were referred to 1 of 4 plastic or reconstructive surgeons at a single academic medical center to discuss BR	60	60	DQI^k^ the 4-item SURE DCS, the BREAST-Q reconstruction module (preoperative; version 1.0), and 3 items from the patient activation measure		2 months		Knowledge, DC, decision process quality, preferences and preference concordance, quality of life, patient activation, and shared decision-making	
Sherman et al [39], Australia	Women diagnosed with BC or ductal carcinoma in situ, who had been advised to undergo and had already undergone a mastectomy, were aged >18 years, were English language competent for reading and writing, had no prior breast surgery (eg, reconstruction or augmentation), and had internet access	116	106	DCS, satisfaction with reconstruction-related information assessed by a 5-item scale, DRS, DASS-21^l^, and SSQ-6^m^		1.6 months		DC, satisfaction with information, and DR	
Varelas et al [31], United States	English-speaking adult women aged >18 years who had been diagnosed with BC (stage I or II only) and had been advised to undergo or had already undergone a mastectomy	13	13	A short knowledge assessment test, STAI, DCS, and BREAST-Q reconstruction module		12 to 18 weeks, 28 weeks, and 36 weeks of pregnancy, and again 6-8 weeks postnatally		Knowledge, psychological status, DC, satisfaction with information provided to a patient by their surgeon, and time of consultation	

^a^BC: breast cancer.

^b^DCS: Decision Conflict Scale.

^c^BR-DMPS: Breast Reconstruction-Decision-Making Process Scale.

^d^DRS: Decision Regret Scale.

^e^BIS: Body Image Scale.

^f^HADS: Hospital Anxiety and Depression Scale.

^g^DC: decisional conflict.

^h^DR: decisional regret.

^i^STAI: State-Trait Anxiety Inventory.

^j^BR: breast reconstruction.

^k^DQI: Decision Quality Index.

^l^DASS-21: Depression Anxiety Stress Scale.

^m^SSQ-6: Social Support Questionnaire.

### Characteristics of the Interventions and Controls

The characteristics of the interventions and controls are shown in Multimedia Appendix 3 [28-31,37-39].

#### Characteristics of the Interventions

In total, 5 of the studies [28-30,38,39] explained that the web-based decision aids development team includes survivors of BC who have undergone mastectomy, plastic or reconstructive surgeons who perform PMBR, and software engineers. The methodology used to develop web-based decision aids includes qualitative research, evidence review and mentoring, and pilot study group meetings. The theoretical basis for the development of web-based decision aids is usually the International Patient Decision Aid Standards [29,30,39] or the Ottawa Decision Support Framework [28]. Except for 2 studies [28,37] that did not report the time of use, most web-based decision aids took between 20 and 74 minutes. Two web-based decision aids [29,30,39] were developed at a reading level written at a seventh- and eighth-grade reading level. The web-based decision aids content specifically includes the patient population and reconstruction options, including implant reconstruction (ie, tissue expanders and implant types), autologous flap reconstruction (ie, latissimus dorsi, rectus abdominis, and free flaps and deep epithelial perforator flaps in the lower abdomen), and skin-sparing and preserving mastectomies (ie, 1-phase and 2-phase procedures). There are also contraindications and general eligibility criteria. Timing of reconstruction includes immediate versus delayed reconstruction, as well as factors that influence the type and timing of reconstruction. It also includes information about the pros and cons of reconstruction versus no reconstruction, implants versus flaps, immediate versus delayed reconstruction, the look and feel of PMBR, and the expected recovery time. The probability of possible implant (eg, wrinkled breast appearance, periosteal contracture after radiation therapy, and possible need for implant replacement over time) and flap (eg, muscle weakness and flap failure) are clearly described in a balanced format with quotes of real patients’ opinions. The web-based decision aids show photographs, high-quality 3D animated images, pre- and postoperative photographs, audio, and video of actual patients of different skin colors and body types, A list of frequently asked questions from clinicians is also included. Elements in the tool include patient-tailored risk assessments, patient value clarification exercises, techniques for managing emotions, and strategies for communicating with family members about PMBR decisions. Women’s stories explaining their reasons for choosing particular methods and their impact on their lives are also included. Users enter their questions and the system prompts them to print a summary to use in a consultation with their physician. This customized printable page also helps patients discuss their concerns and options with their families.

#### Characteristics of the Controls

The control for the study by Politi et al [30] was the enhanced urgent care and American Society of Plastic Surgeons pamphlet on PMBR. Varelas et al [31] used traditional counseling. The control for the study by Fang et al [28] was the provider-provided urgent care+pamphlet, which describes the types of surgery, including mastectomy, implant-based PMBR, and autologous PMBR, as well as the advantages and disadvantages of the different types of surgeries. The control for the study by Manne et al [38] was the 56-page pamphlet available at no cost from the Cancer Support Community focusing on PMBR. For the study by Sherman et al [39], the control was the web-based access to excerpts of the public brochure, including basic information on breast surgery and reconstruction, but excluding content unique to the intervention group (ie, video interviews with patients or surgeons, and values clarification exercises). In the study by Mardinger et al [29], the control was the decision aids, which is unvalidated that contains 6 text-based pages that can be accessed in both interactive and noninteractive formats. The control for the study by Heller et al [37] was the group that received the standard patient education, including printed materials in books and pamphlets as well as personal instruction from the attending physician, physician-in-training, physician assistant, and nurse practitioner.

### Outcome Measure

A total of 5 studies [28,29,31,38,39] measured decision conflict using the Decision Conflict Scale (DCS), and 1 study [30] measured decision conflict using the 4-item SURE scale. Three studies [28,29,39] measured decision regret using the Decision Regret Scale (DRS) and 2 studies [28,29] measured informed choice using the subdimension of the DCS—feeling informed. Knowledge was measured primarily by the percentage of correct answers to self-administered multiple-choice questions about specific plastic surgery procedures in 6 studies [28-31,37,38]. Satisfaction was measured using the Satisfaction with Decision Scale [29] and some scales adapted from those used in previous studies [28,37-39]. Anxiety was primarily measured using the Hospital Anxiety and Depression Scale [28] and the State-Trait Anxiety Inventory [31,38].

### Decision-Related Outcomes

#### Decision Conflict

**Figure 2:**
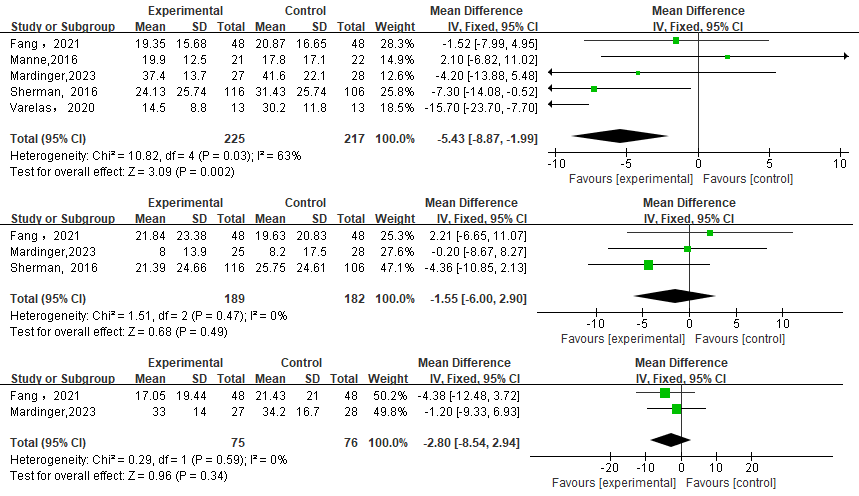
Forest plot comparing decision conflict [28,29,31,38,39], decision regret [28,29,39], and informed choice [28,29] in the web-based decision aids group versus control groups.

In total, 6 studies [28-31,38,39] investigated the impact of decision conflicts in PMBR. The 5 studies [28,29,31,38,39] that used DCS included in the meta-analysis showed a statistically significant positive impact of web-based decision aids interventions on decision conflict (MD=–5.43, 95% CI –8.87 to –1.99; *P*=.002). Heterogeneity experiments indicated that there was evidence of statistical heterogeneity in the expected summary results (*I*^2^=63%; Figure 2). Politi et al [30] used the 4-item SURE DCS and reported that there was no difference between the 2 groups in terms of decisional conflict (*P*>.05).

#### Decision Regret

In total, 3 studies [28,29,39] used DRS to investigate the impact of decision regret in PMBR. The meta-analysis showed that the difference in decision regret after the intervention was not statistically significant compared with the control group (MD=–1.55, 95% CI –6.00 to 2.90; *P*=.49). Heterogeneity experiments indicated that there was evidence of no statistical heterogeneity in the expected summary results (*I*^2^=0%; Figure 2).

#### Informed Choice

In total, 2 studies [28,29] investigated the impact of informed choice by DCS in PMBR surgery. The meta-analysis showed that the difference in informed choice after the intervention was not statistically significant compared to the control group (MD=–2.80, 95% CI –8.54 to 2.94; *P*=.34). Heterogeneity experiments indicate that there was evidence of no statistical heterogeneity in the expected summary results (*I^2^*=0%; Figure 2).

#### Knowledge

We did not conduct a meta-analysis of knowledge as an outcome because most of the instruments measuring knowledge were self-administered. The study by Heller et al [37] found significantly higher levels of knowledge in the web-based decision aids group, with a mean increase in correctly answered questions of 14% compared to 8% in the control group (*P*=.02). Politi et al [30] found that participants using web-based decision aids had higher objective knowledge, answering an average of 85% (9.35/11) of the questions correctly compared to 58% (6.35/11) in the control group (*P*<.001). Similarly, Varlas et al [31] showed improved knowledge assessment scores in both groups but significantly higher knowledge assessment scores in the intervention group (control=70.8%, SD 15.5%; intervention=83.1%, SD 13.8%; *P*=.02). However, Manne et al [38] reported similar effects of web-based decision aids on PMBR knowledge versus the booklet, and Fang et al [28] also reported no difference in the amount of PMBR-related medical information between web-based decision aids and the control group at 1 week after consultation (*P*=.13), suggesting that women in both groups had a similar level of comprehension of medical information, whether using the booklet alone or in combination with the web-based decision aids. Mardinger et al [29] also reported that both groups had similar scores on the true or false PMBR knowledge questionnaire over time (*P*>.05).

### Psychological Outcomes

#### Satisfaction

**Figure 3:**
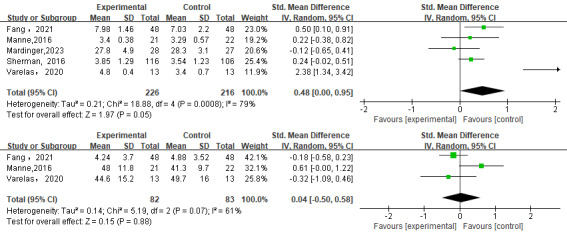
Forest plot comparing satisfaction [28,29,31,38,39] and anxiety [28,31,38] in the web-based decision aids group versus control groups.

In total, 5 studies [28,29,31,38,39] used different scales to investigate the impact of satisfaction. The meta-analyses indicated that web-based decision aids may improve current form:satisfaction compared to controls, but the results were not statistically significant (SMD=0.48, 95% CI 0.00 to 0.95; *P*=.05). Heterogeneity experiments indicated that there was evidence of statistical heterogeneity in the expected summary results (*I^2^*=79%; Figure 3). Similarly, Heller et al [37] reported a higher level of satisfaction with the way in which information about PMBR was obtained in the web-based decision aids group than in the control group (*P*=.03).

#### Anxiety

A total of 3 studies used Hospital Anxiety and Depression Scale and State-Trait Anxiety Inventory [28,31,38] to investigate the impact of anxiety in PMBR. The meta-analysis showed that there was no statistically significant difference in the combination of SMD after intervention (SMD=0.04, 95% CI –0.50 to 0.58; *P*=.88). Heterogeneity experiments indicated that there was evidence of statistical heterogeneity in the expected summary results (*I^2^*=61%; Figure 3). Heller et al [37] reported that in the web-based decision aids group, there was a trend toward lower levels of anxiety between the preoperative and postoperative visits, but the difference between the groups was not significant as determined by generalized estimating equation modeling.

#### Choice of Surgery

The surgical choices differed between the two groups in the study by Fang et al [28]: 56% (27/48) in the web-based decision aids group and 46% (22/48) in the control group opted for immediate PMBR (*P*=.05). In addition, most patients chose implantable PMBR, with no difference between groups. Notably, the web-based decision aids group in the study by Mardinger et al [29] was unbalanced in terms of the choice of type of PMBR, with 10 (36%) women in the web-based decision aids group refusing PMBR compared with 6 (21%) women in the control group (*P*=.20). The results of the study by Politi et al [30] showed that 95 (79.2%) women underwent reconstruction; among them, nearly all (92/95, 97%) underwent immediate PMBR, and there were no differences between groups in median preference scores for reconstruction, type, or time.

### Evaluation of the Intervention

In total, 3 studies reported different benefits of web-based decision aids compared to controls. Heller et al [37] reported an upward trend in the number of patients in the web-based decision aids group who reported that they received all the necessary information and improved their ability to choose a PMBR plan, but the difference between the groups was not significant. Manne et al [38] reported that 81% of participants in the web-based decision aids found logging in and navigating easy and the length of time was rated as “just right,” and that the web-based decision aids were more helpful, interesting, and valuable than the brochures. Sherman et al [39] found that women in the intervention group found the web-based decision aids to be 2.94 (SD 0.76) informative, very useful, easy to use, contained enough information, and helped them to clarify their reconstruction ideas. However, Varelas et al [31] reported that surgeon satisfaction was also significantly higher in the intervention group than in the control group. Meanwhile, consultation time was shorter in the intervention group, but the difference was not statistically significant (*P*=.46). Similarly, Politi et al [30] reported no difference between the web-based decision aids group and the control group in terms of mean counseling time after the intervention (29.7 vs 30.0 minutes; *P*>.05). Mardinger et al [29] showed that although women used both decision aids with comparable frequency, the total time spent counseling and the time spent per counseling session was significantly greater for women in the intervention than that for the control group (*P*<.05). Women in the study by Fang et al [28] indicated no difference between the 2 groups in terms of perceived impact and utility of web-based decision aids on PMBR decisions.

### Sensitivity Analysis

**Figure 4:**
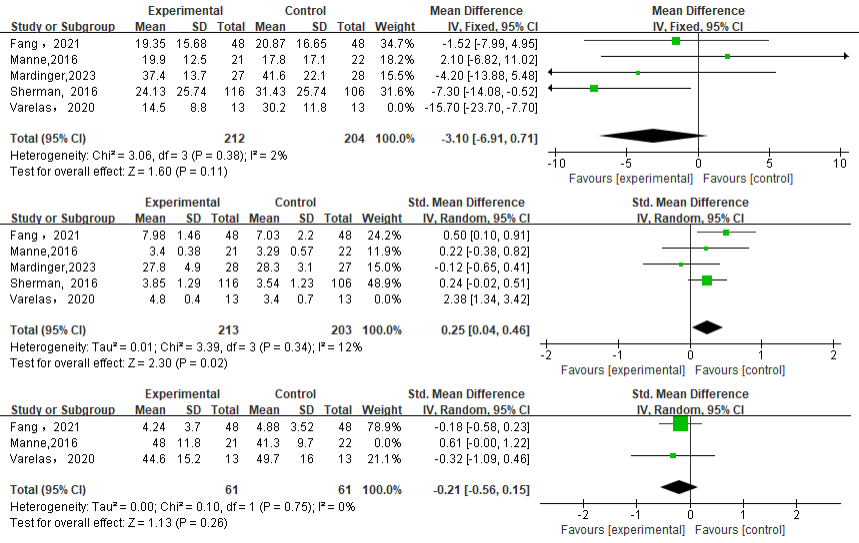
Sensitivity analysis of decision conflict [28,29,38,39], satisfaction [28,29,38,39], and anxiety [28,31].

We conducted sensitivity analyses of decision conflict, satisfaction, and anxiety by removing each study. Sensitivity analysis showed that for decision conflict and satisfaction, after removing 1 study [31], contrary to the previous results, web-based decision aids did improve satisfaction (the *I^2^* range was 79%-12%) but did not improve decision conflict (the *I^2^* range was 63%-2%). We found that by removing the study by Manne et al [38], the stability of anxiety did not change but the heterogeneity was reduced from 62% to 0% (Figure 4).

### Risk of Bias

**Figure 5:**
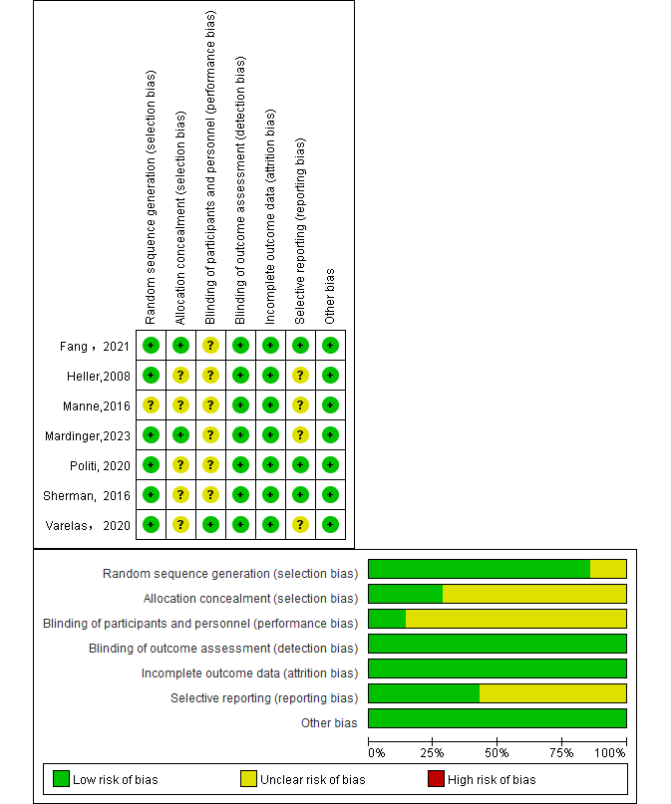
Risk of bias assessment of the included studies [28-31,37-39].

Figure 5 [28-31,37-39] presents the summary of the risk of deviation for the included studies. In 6 [28-31,37,39] of the 7 studies, the description of the method used in random assignment was clearly stated (ie, web-based automated randomization software and random number generator), and in the remaining study [38], the information obtained about random assignment was insufficient to make a definitive judgment. Of the 7 studies, 5 [30,31,37-39] were unable to make definitive judgments in this area because of underreporting, whereas in the remaining 2 trials [28,29] sufficient information was obtained about allocation concealment (individually sealed envelopes to conceal allocation). Furthermore, 6 studies [28-30,37-39] were judged to be at unclear risk of bias because the effect of unblinding was unknown, and 1 study [31] described the blinding of participants. Seven studies [28-31,37-39] achieved blinding of outcome evaluators (ie, clinic and surgical staff were blinded to condition assignment) or the blinding was unclear, but the outcome was objectively measured and not subjective to interpretation. Incomplete outcome data appeared to be adequately addressed in 7 studies [28-31,37-39] (ie, incomplete data were fairly evenly balanced across intervention groups or intention-to-treat analyses were reported). In addition, 3 studies [28,30,39] underwent clinical registration or reported relevant protocols, showing that outcomes were reported in full. The impact of selective reporting in the remaining 4 studies [29,31,37,38] was unclear, and this area was judged to be at unclear risk of bias. Information on other potential sources of bias was sufficient. Therefore, this area was judged to be at low risk of bias for all studies [28-31,37-39].

### Certainty of Evidence

We assessed the certainty of evidence for the included RCTs using Grades of Recommendation Assessment, Development, and Evaluation (GRADE; Multimedia Appendix 4) except for decision regret, for which the certainty of evidence was low. The certainty of evidence was very low for the rest, that is, decision conflict, satisfaction, anxiety, and informed choice.

## Discussion

### Principal Findings

Our systematic review and meta-analysis showed that the modules of web-based decision aids include basic information on PMBR, patient stories, risk assessment, value clarification, and emotion management and that patients can be directed to seek information and obtain personalized decision support based on their individual needs. Therefore, these web-based decision aids are helpful and recommended for women. Regarding the effectiveness of web-based decision aids, the results showed that they may improve PMBR knowledge, decision conflict, and satisfaction but have no effect on informed choice, decision regret, or anxiety. The overall GRADE quality of evidence for decision regret was low, and the overall GRADE quality of evidence for informed choice, decision conflict, and anxiety was very low.

### The Content of Web-Based Decision Aids

First, regarding the content of web-based decision aids, few of the studies included in our systematic review and meta-analyses reported comprehensive development of their web-based decision aids. The types of decisions on which most web-based decision aids primarily focused were PMBR decision types and reconstruction times. In addition, some of the studies reported that the development of the tool was obtained through a decisional needs assessment. Research suggests that people tend to have decisional needs when confronted with known outcomes with multiple choices, uncertain outcomes, or valuing people differently and that unmet needs lead to poor quality decisions, which adversely affect health outcomes [40]. Research has shown that some patients have difficulty imagining plastic surgery without photos of women of different body types and skin colors when faced with a decision. Therefore, the use of 3D images during the counseling process is an acceptable web-based decision aid, and the results of our review suggest that web-based decision aids on PMBR decision-making show real photographs of patients by incorporating high-quality, 3D animated images and that viewing 3D images may increase presurgical preparation by giving patients a more realistic understanding of what is actually achievable after PMBR [41]. There are web-based decision aids that are designed with the goal of making patients more comfortable receiving information in a less-stressful environment outside of the hospital, and it also allows family members and friends who are members of the patient support group, but who may not necessarily be able to participate in the counseling, to receive specific information about the procedure and participate in the decision-making process. Women and their families are allowed to express their views about breast surgery because family members act as advocates and care coordinators in the decision-making process [42]. In this era of increasing emphasis on evidence-based medicine, the PMBR risk assessment calculator can help individualize and quantify risk to better inform surgical decisions and better manage patient expectations [43]. The purpose of the values clarification exercise is to help women assess, explore, and identify their personal values and to encourage them to think about how their values affect their decision-making. Using the values clarification exercise can help women increase their satisfaction with their appearance. Patient stories are also important to web-based decision aids, and research has shown that women express a need to learn about other women’s experiences to gain a deeper understanding of the impact of PMBR on their daily lives. Web-based decision aids have achieved this by telling the stories of patients who have had previous mastectomies, with or without PMBR. These stories illustrate the decision-making experiences of these patients and the impact of their decisions on their daily lives [44]. Another advantage of web-based decision aids is that they allow patients to absorb the information without being overwhelmed by other information or distracted by other issues. Research has shown that some people feel prepared and emotionally supported for PMBR decision-making, while others feel that the elements of supportive care are missing, making the inclusion of an emotion management module in web-based decision aids essential for women’s psychosocial support [45]. However, although the internet has become an easily accessible tool, there is still a persistent digital divide. Therefore, special attention should be given to the sociodemographic characteristics of the population, building more resources for health care infrastructure in underserved communities and providing free or discounted Wi-Fi connections and mobile devices in low-income areas [46]. These actions, combined with the popularity of smartphone users, are measures that may narrow the digital divide [21].

### Effectiveness of Web-Based Decision Aids

In line with the results of a previous meta-analysis [26], web-based decision aids reduced decision conflict. Decision conflicts were as high as 45.68 (SD 23.40) among women who were newly diagnosed with early-stage BC in China [47]. Decision conflict was significantly higher among women who chose mastectomy with or without combined reconstruction compared to women who chose conservative breast surgery. Greater decision conflict is associated with less information, higher uncertainty in weighing choices based on personal values, and inadequate social support [40]. Women may second-guess their decisions after the fact, even if those decisions have already been made. Women who face PMBR decision-making need support in making this complex decision, especially those who do not have a strong preference for PMBR. Decision conflict can be reduced by addressing factors of uncertainty, such as providing information about the benefits and risks of each option and helping patients understand their own values [48]. Web-based decision aids can improve the quality of PMBR decision-making by enhancing patient knowledge and providing personalized risk assessments, reducing decision conflict [18].

Uncertainty about whether they are making the best decision can trigger emotional turmoil, and decision regret occurs when women compare the unfavorable outcome of a decision with alternative choices they may have [11,47]. The results of our meta-analysis showed that there was no effect of web-based decision aids on decision regret in the intervention group compared to the control. Women who choose decisions that result in unexpected clinical outcomes or lower-than-expected outcomes will inevitably experience decision regret, a very common but negative emotion, even though the patient’s preferences and needs are honored and considered in their treatment [49]. Decision regret can be used as an indicator of decision-making quality, which can contribute to performance improvement in the health care system. Other studies from a psychological perspective have shown that if a decision is regretted, the following “preference reversal” may cause patients to favor another unselected option, which may completely offset their health outcomes, with the degree of decision regret varying widely. However, Becerra Pérez et al [50] reported that most studies reported a low mean DRS, resulting in an overall mean score of 16.5 out of 100 across studies. It is important to note that there is no consensus on specific thresholds for clinically important decision regret based on DRS, and authors have rarely justified their choice of thresholds; therefore, minimum and maximum efficiency may limit our ability to perform statistical analyses [51].

Previous research has shown that women with BC who use decision aids receive more information that helps them make informed and values-based decisions [26]. Our results, in contrast, showed no effect of web-based decision aids on informed choice in the intervention group compared to the control group possibly because, compared to other forms, web-based decision aids require more effort. Therefore, some women in the web-based decision aids group may have been less inclined to seek more information and consider it carefully. This may explain why women in the web-based decision aids group did not feel less uninformed about their decisions [52]. The results of previous meta-analyses [25,26] suggest that web-based decision aids are promising interventions for improving knowledge related to PMBR decision-making and that web-based decision aids can help patients’ knowledge of PMBR and treatment options and can identify patients’ PMBR preferences and goals for quality decision-making with their health care providers; however, it is important to note that in this review, the impact of web-based decision aids on PMBR knowledge was mixed, which may be because most of the current instruments on PMBR decision-making knowledge measurement are self-administered scales. We found that web-based decision aids improved PMBR knowledge compared to a control group of some conventional education [37], traditional counseling [31], or conventional pamphlets [30]. When the control group was using pamphlets [19,28] or noninteractive decision aids [29] that contained similar information, web-based decision aids did not have a statistically significant effect on PMBR knowledge. Therefore, to elucidate the impact of web-based decision aids on knowledge, measurement studies using validated and sensitive instruments are needed.

Because the initial anxiety experienced by women may be related to the new diagnosis and anticipated surgery, this anxiety lessened once the surgery was over. There was no difference in the level of anxiety experienced after surgery between the 2 groups. Given the severity of a BC diagnosis, it is very reassuring that web-based decision aids did not exacerbate anxiety while providing benefits in terms of patient satisfaction and knowledge as well as surgeon satisfaction. Several studies have shown that patient satisfaction is higher when receiving PMBR information digitally [53]. Our study also suggests that web-based decision aids improve patient satisfaction with decision-making. Although most of the studies included in our systematic review reported that the use of web-based decision aids increased women’s satisfaction with PMBR, most of the measurement tools used to assess the outcomes used self-administered scales. Therefore, more high-quality evidence, including studies using validated and sensitive instruments, is needed to elucidate the impact of web-based decision aids on satisfaction [26].

### Sensitivity Analysis

Some of the outcome indicators in this review (ie, decision conflict, satisfaction, and anxiety) showed significant heterogeneity, which may be related to factors such as the fact that the measurement tools were different and the web-based decision aids were delivered in an inconsistent form and content. We conducted sensitivity analyses for decision conflict, satisfaction, and anxiety, and the adjusted total estimates of anxiety did not significantly change these results when studies were progressively omitted, excluding the study by Manne et al [38]. With respect to decision conflict and satisfaction, the adjusted total estimates changed significantly, a result that excludes the study by Varelas et al [31]. Contrary to the original results, the effect of web-based decision aids on improving satisfaction was statistically significant, and the effect of web-based decision aids on improving decision conflict was similar to the control group effect; therefore, the effect of web-based decision aids on decision conflict and satisfaction should be carefully interpreted. Regarding the heterogeneity of this meta-analysis, sensitivity analyses showed that the heterogeneity of all outcomes was also reduced by excluding 1 study.

### Limitations

Some limitations of this review must be recognized. First, we did not perform an assessment of publication bias because only 7 studies were ultimately included in the analysis, which may cause publication bias. In addition, the included studies had no follow-up surveys and lacked evidence of the long-term impact of the interventions. Our findings serve as a reminder that even when statistical information is effectively communicated, participants may not make estimates of the same order of magnitude after a period. Finally, the number of included studies was small. Some studies had inconsistent outcome indicators and were therefore not included.

### Conclusions

This review shows that web-based decision aids can increase knowledge and satisfaction, and reduce levels of decision conflict among women facing PMBR decision-making; however, there is no effect on informed choice, decision regret, or anxiety. Currently, web-based decision aids for women’s PMBR decision-making are relatively easy to implement in terms of content and form. Due to limitations in the number of included studies in our meta-analysis, well-designed studies, including multicenter RCTs using high-quality decision aids, are necessary in the future to further validate our conclusion that web-based decision aids play a role in the quality of decision-making for women facing PMBR.

## Appendix

Multimedia Appendix 1 PRISMA (Preferred Reporting Items for Systematic Reviews and Meta-Analyses) statement.

Multimedia Appendix 2 Search strategy.

Multimedia Appendix 3 Characteristics of the interventions and controls.

Multimedia Appendix 4 Certainty of evidence.

## Abbreviations

BC: breast cancer
DCS: Decision Conflict Scale
DRS: Decision Regret Scale
GRADE: Grades of Recommendation Assessment, Development, and Evaluation
MD: mean difference
PMBR: postmastectomy breast reconstruction
PRISMA: Preferred Reporting Items for Systematic Reviews and Meta-Analyses
RCT: randomized controlled trial
SMD: standardized mean difference
