# Supplementary material for: Assessing the Content and Effect of Web-Based Decision Aids for Postmastectomy Breast Reconstruction: Systematic Review and Meta-Analysis of Randomized Controlled Trials
Source: J Med Internet Res. 2024 May 27;26:e53872. doi: 10.2196/53872 (PMC11165285; doi:10.2196/53872)
Supplement: Multimedia Appendix 2 [file jmir_v26i1e53872_app2.docx]

**Search strategy**

**Pubmed**

((((("Mastectomy"[Mesh]) OR "Mammaplasty"[Mesh]) OR (mastectom*[Title/Abstract] OR mammectom*[Title/Abstract] OR mammaplast*[Title/Abstract] OR mastoplast*[Title/Abstract] OR breast reconstruct*[Title/Abstract])) AND ((randomized controlled trial[Publication Type] OR controlled clinical trial[Publication Type]) OR (randomized[Title/Abstract] OR trial[Title/Abstract] OR placebo[Title/Abstract] OR randomly[Title/Abstract]))) AND ((((((("Decision Trees"[Mesh]) OR "Decision Support Techniques"[Mesh]) OR "Decision Support Systems, Clinical"[Mesh]) OR "Audiovisual Aids"[Mesh]) OR "Decision Making"[Mesh]) OR "Choice Behavior"[Mesh]) OR (decision* aid*[Title/Abstract] OR decision* support*[Title/Abstract] OR decision* tool*[Title/Abstract] OR decision* instrument*[Title/Abstract] OR decision* technolog*[Title/Abstract] OR decision* technique*[Title/Abstract] OR decision* system*[Title/Abstract] OR decision* program*[Title/Abstract] OR decision* algorithm*[Title/Abstract] OR decision* process*[Title/Abstract] OR decision*method*[Title/Abstract] OR decision* intervention*[Title/Abstract] OR decision* material*[Title/Abstract] OR risk communication tool*[Title/Abstract] OR risk assessment tool*[Title/Abstract] OR risk information tool*[Title/Abstract] OR risk communication method*[Title/Abstract] OR risk assessment method*[Title/Abstract] OR risk information method*[Title/Abstract] OR patient decision*[Title/Abstract] OR informed decision*[Title/Abstract] OR informed choice*[Title/Abstract] OR shared decision making[Title/Abstract])) ) AND (((interactive[tw] OR computer*[tw] OR web based[tw] OR Internet[Mesh] OR User-Computer Interface[Mesh] OR Decision Making, Computer-Assisted[Mesh] OR medical informatics [Mesh]))))

**Web of science**

TS=(random* controlled trial OR controlled clinical trial OR random* OR trial OR placebo )

TS=(Animal* NOT ("Human* AND "Animal*))

TS=( Mastectomy OR Mammaplasty OR mastectom* OR mammectom* OR mammaplast* OR mastoplast* OR breast reconstruct*)

TS=(Decision Making OR Choice Behavior )

TS=(Decision Tree* OR Decision Support Technique* OR Decision Support Systems, Clinical OR Audiovisual Aid*)

TS=(decision* aid* OR decision* support* OR decision* tool* OR decision* instrument* OR decision* technolog* OR decision* technique* OR decision* system* OR decision* program* OR decision* algorithm* OR decision* process* OR decision*method* OR decision* intervention* OR decision* material* OR risk communication tool* OR risk assessment tool* OR risk information tool* OR risk communication method* OR risk assessment method* OR risk information method* OR patient decision* OR informed decision* OR informed choice* OR shared decision making)

TS= (((interactive OR computer* OR web based OR Internet OR User-Computer Interface OR Decision Making, Computer-Assisted OR medical informatics ))))

**Cochrance**

1. Decision Support Techniques Single MeSH term (unexploded)
2. Decision Support Systems, Clinical Single MeSH term (unexploded)
3. decision trees Single MeSH term (unexploded)
4. decision making Single MeSH term (unexploded)
5. choice behavior Single MeSH term (unexploded)
6. audiovisual aids Single MeSH term (unexploded)
7. (decision* NEAR/3 support*) OR (decision* NEAR/3 aid*) OR (decision* NEAR/3 tool*) OR (decision* NEAR/3 instrument*) OR (decision* NEAR/3 technolog*) OR (decision* NEAR/3 technique*) OR (decision* NEAR/3 system*) OR (decision* NEAR/3 program*) OR

(decision* NEAR/3 algorithm*) OR (decision* NEAR/3 process*) OR (decision* NEAR/3 method*) OR (decision* NEAR/3 intervention*) OR (decision* NEAR/3 material*)

1. (“risk communication” NEAR/3 tool*) or (“risk communication” NEAR/3 method*) or (“risk information” NEAR/3 tool*) or (“risk information” NEAR/3 method*) or (“risk assessment” NEAR/3 tool*) or (“risk assessment” NEAR/3 method*)
2. shared decision making
3. informed choice*
4. informed decision*
5. patient decision*
6. (risk communication” NEAR/3 tool*) or (“risk communication” NEAR/3 method*) or (“risk information” NEAR/3 tool*) or (“risk information”NEAR/3 method*) or (“risk assessment” NEAR/3 tool*) or (“risk assessment” NEAR/3 method*): ti,ab,kw

14 MeSH descriptor: [Mastectomy] this term only

15MeSH descriptor: [Mammaplasty] this term only

16 mastectom*

17 mammectom*

18 mammaplast*

19 mastoplast*

20 breast reconstruct*

**Embase**

#11:#7 OR #8 OR #9 OR #10

#10:'decision making'/exp

#9:'shared decision making':ab,ti OR 'informed choice*':ab,ti OR 'informed decision*':kw OR 'patient decision*':ab,ti

#8:'risk communication tool*':ab,ti OR 'risk information method*':ab,ti OR 'risk communication method*':ab,ti OR 'risk information tool*':ab,ti OR 'risk assessment method*':ab,ti OR 'risk assessment tool*':ab,ti

#7:'decision support system'/exp OR 'clinical decision support system'/exp OR 'decision tree'/exp OR 'audiovisual aid'/exp

#4:'randomized controlled trial':it OR trial:ti,ab,kw OR randomized:ti,ab,kw OR 'controlled clinical trial':it OR randomly:ti,ab,kw OR placebo:ti,ab,kw

Pregnancy

birth OR cesarean OR vaginal birth after cesarean OR delivery, Cesarean Section
