# Supplementary material for: Assessing the Content and Effect of Web-Based Decision Aids for Postmastectomy Breast Reconstruction: Systematic Review and Meta-Analysis of Randomized Controlled Trials
Source: J Med Internet Res. 2024 May 27;26:e53872. doi: 10.2196/53872 (PMC11165285; doi:10.2196/53872)
Supplement: Multimedia Appendix 3 [file jmir_v26i1e53872_app3.docx]

^Table: Characteristics of interventions and control^

| **Study, year, country** | **Intervention** | | | | | | | **Control** |
| --- | --- | --- | --- | --- | --- | --- | --- | --- |
|  | **Format** | **Theory** | **Content** | **Duration** | **Development team** | **Development method** | **reading level** |  |
| Fang et al, 2020[28], China | Application | ODSF^a^ | Information regarding surgical options(breast reconstruction and mastectomy, such as the advantages and disadvantages, the complication probabilities of each option, a value clarification exercise for the patient’s self-evaluation, and a summary of the participant’s decision-making process. | NR^b^ | Software engineer,  breast surgeon, breast care nurses,  informaticist,  a researcher specializing in breast cancer. | Literature reviews and related websites. | NR | Usual care from health care providers and pamphlet with information about types of surgery, including mastectomy, implant-based BR, and autologous BR, and the advantages and disadvantages of different surgery types. |
| Heller et al,  2008[37], United States | Single compact disk | None | High-quality, three-dimensional animated graphics, patient testimonials, before-and-after photographs, and video explanations from plastic surgeons and clinical specialists in surgical, medical, and radiation oncology. | NR | University of Texas M. D. Anderson Cancer Center | Focus groups and faculty discussions. | NR | Standard patient education. |
| Manne et al, 2015[38], United States | Menu-driven program organized into 10 modules | None | Introductory tour, users encounter a home menu page that lists modules addressing an identified area of need for women considering BR. | 74min | Breast Cancer Surgeon, Reconstructive Surgeon, Breast Cancer Oncologist, Research Principal Investigator, Software Company Triad Interactive, Inc., Female. | Team meetings and discussions, in-depth interviews. | NR | A free 56-page Cancer Support Community brochure. |
| Mardinger, et al[29], 2023, Australia | Menu-driven modular format. | IPDAs^c^, psychological theoretical models | Provides information on breast cancer surgery and reconstructive options, including illustrations, associated risks, benefits and possible complications; video interviews and photo galleries of patients/surgeons, with value clarification and stress management. | 45min | A multidisciplinary advisory team (representing surgery, radiation oncology, medical oncology, nursing and women with breast cancer) | Literature review, consultations. | 8th grade level | Unvalidated decision aid containing six text-based pages accessible in both interactive and non-interactive formats. |
| Politi et al,  2020[30], United States | Interactive learning modules | IPDAs | Tailored risk assessments covering general information about reconstruction and decision-making options, balanced tables in the tool listing the pros and cons of each option with quotes from real patients' opinions, displaying photos of real patients of different skin colours and sizes, and users typing in their own questions and printing summaries for use during consultations. | 26.7min | Qualitative input from stakeholders including 20 breast cancer survivors who had undergone mastectomy, 10 plastic/reconstructive surgeons who perform breast reconstruction, and 10 nurses who treat breast reconstruction patients. | Qualitative study, vidence review and guidance, pilot study. | The content was written at a 7th grade reading level. | American Society of Plastic Surgeons pamphlet on breast reconstruction. |
| Sherman et al[39], 2016, Australian | Menu-driven modular format | IPDAs, psychological theoretical models | Provides information on breast cancer surgery and reconstructive options, including illustrations, associated risks, benefits and possible complications; video interviews and photo galleries of patients/surgeons, with value clarification and stress management. | 45min | A multidisciplinary advisory team (representing surgery, radiation oncology, medical oncology, nursing and women with breast cancer) | Literature review, consultations | 8th grade level | Excerpted information from the publicly available Women's Guide to Early Breast Cancer, including basic information about breast surgery and reconstruction. |
| Varelas et al[31], 2020, United States | Decide program | None | Evidence-based information, explicit probability illustrations, and values clarification exercises. | NR | Approximately 20 minutes | NR | NR | Traditional consultation. |

^a^ODSF: Ottawa Decision Support Framework

^b^NR: Not report

^c^IPDAS: International Patient Aids Stadand
