# Supplementary material for: Assessing the Content and Effect of Web-Based Decision Aids for Postmastectomy Breast Reconstruction: Systematic Review and Meta-Analysis of Randomized Controlled Trials
Source: J Med Internet Res. 2024 May 27;26:e53872. doi: 10.2196/53872 (PMC11165285; doi:10.2196/53872)
Supplement: Multimedia Appendix 4 [file jmir_v26i1e53872_app4.docx]

Table the quality of evidence.

| Certainty assessment | | | | | | | N of patients | | Effect | Certainty | Importance |
| --- | --- | --- | --- | --- | --- | --- | --- | --- | --- | --- | --- |
| N of studies | Study design | Risk of bias | Inconsistency | Indirectness | Imprecision | Other considerations | Interventiongroup | control group | Absolute  (95% CI) |  |  |
| Decision Conflict | | | | | | | | | | | |
| 5 | randomised  trials | serious  ^a^ | serious  ^b^ | not serious | serious  ^c^ | none | 225 | 217 | MD -5.43 lower (-8.87 lower to -1.99 lower) | ⨁◯◯◯ Very low | CRITICAL |
| Decision regret | | | | | | | | | | | |
| 3 | randomised  trials | serious  ^d^ | not serious | not serious | Very serious  ^e^ | none | 189 | 182 | MD -1.55 lower  (-6.00 lower to 2.90  higher) | ⨁⨁◯◯  Low | CRITICAL |
| Satisfaction | | | | | | | | | | | |
| 5 | randomised  trials | serious  ^d^ | not serious | not serious | serious^f^ | none | 226 | 216 | SMD 0.48 lower (0.00 lower to 0.95 lower) | ⨁◯◯◯ Very low | CRITICAL |
| Anxiety | | | | | | | | | | | |
| 3 | randomised  trials | serious  ^d^ | serious  ^b^ | not serious | serious  ^e^ | none | 82 | 83 | SMD 0.04 lower (-0.50 lower to 0.58 lower) | ⨁◯◯◯ Very low | CRITICAL |
| Informed choice | | | | | | | | | | | |
| 2 | randomised  trials | serious  ^d^ | serious  ^g^ | not serious | serious  ^e^ | none | 75 | 76 | MD -2.8 lower  (-8.54 lower to  2.94 higher) | ⨁◯◯◯ Very low | CRITICAL |

CI: confidence interval; MD: mean difference; SMD: standardised mean difference

Explanations

1. Allocation concealment and blinding in research Risks not clear, the contribution of studies that studies that are inconsistent with results of the sensitivity analysis is significant
2. Downgraded one level for inconsistency due to unexplained variation between studies (Chi²test statistically significant and I² > 50%).
3. Quality of evidence reduced by one level due to significant imprecision caused by a sample size of less than 400 participants
4. Allocation concealment and blinding in research risk not clear
5. Certainty of the evidence was downgraded by two levels due to very serious imprecision: The 95% cl has a very wide range; fewer than 400 participants
6. Use several scales to measure outcomes
7. Specific scales not used
